# An Oil Slick Detection Method Based on Advanced Spectral DNA Encoding Strategy by Chinese Zhuhai-1 Satellite Imagery

**DOI:** 10.3390/s26123954

**Published:** 2026-06-22

**Authors:** Dong Zhao, Lihui Bi, Jianqiao Feng, Guoxiang Gao, Chuang Qu

**Affiliations:** 1 Key Laboratory of Exploration Technologies for Oil and Gas Resources (Yangtze University), Ministry of Education, Wuhan 430100, China; zhaodong@yangtzeu.edu.cn (D.Z.); 18171341596@163.com (G.G.);; 2 Hezhou Natural Resources Bureau, Hezhou 542800, China; hzskbk@163.com

**Keywords:** Chinese Zhuhai-1 satellite, marine oil spill, hyperspectral remote sensing, spectral encoding, DNA encoding

## Abstract

In recent years, wars have gradually increased the risk of marine oil spill accidents. Marine oil spill monitoring becomes more and more important for preventing marine oil pollution. The Chinese Zhuhai-1 satellite can capture abundant spectral reflectance signals. It is a significant way of detecting marine oil spills. Most of the traditional oil spill detection methods only used a small amount of spectral information. It made it difficult identify oil spills accurately from the inhomogeneous marine environment. In order to mine the key differential spectral information of oil slicks, inspired by the encoding method of spectral DNA, an advanced spectral DNA encoding (ASDE) strategy was proposed to describe the spectral details in Zhuhai-1 images. On this basis, two kinds of key spectral information extraction methods were proposed to mine the spectral genes of oil slicks. Finally, the extracted spectral genes were used to detect the marine oil spills. Three Zhuhai-1 satellite images were used to validate the performance of the proposed method based on ASDE strategy. The experimental results indicated that the proposed method could precisely describe the spectral differences in oil slicks and seawater in Zhuhai-1 images. In addition, the extracted spectral genes could detect marine oil spills correctly.

## 1. Introduction

With the continuous development of global societies and economies, the demand for crude oil is expanding. More marine oil transportation and exploitation activities lead to increased risk of oil spills [1,2]. At present, many wars around the world make oil spill accidents inevitable. Thus, it becomes very necessary to monitor the oil spills at sea [3,4].

Hyperspectral satellites acquire many spectral reflection signals of surface targets over a wide range. They can produce hyperspectral images (HSI) for identifying and monitoring marine oil spills [5,6]. Studies on marine oil spill detection methods based on HSI help to prevent and control the pollutions. Most of the current satellite oil spill detection methods are based on images captured by Sentinel-1/2, Hyperion, MODIS, Proba, etc. [7,8,9,10]. The Chinese Zhuhai-1 (02) hyperspectral satellite can get images of 32 bands and the Zhuhai-1 (03) hyperspectral satellite can get images of 256 bands [11,12]. Zhuhai-1 satellite imagery gains the advantages of wide coverage and short revisit period. It is a favourable way of monitoring marine oil spills [13,14]. In this research, the oil spill detection method is based on Zhuhai-1 satellite (02) imagery.

The main way to identify marine oil slicks caused by spill accidents is mining the distinctive spectral signals in HSI. Many innovative studies have been proposed in this field. In 2003, Carl Brown and Mervin Fingas proposed that the spectral reflection signals of the surface oil slicks in the infrared bands were higher than that of the background seawater. The infrared spectral reflection signals could be used to identify marine oil spills [15]. In 2011, Yingcheng Lu et al. proposed a decision tree method of spectral characteristic bands to identify marine oil spills based on HJ-1 satellite imagery. This method performed well in the Gulf of Mexico oil spill remote sensing data [16]. In 2012, Eduardo Loos et al. demonstrated that the ability of fluorescence index (FI) and rotation absorption index (RAI) to distinguish oil slicks and “false targets” through MODIS satellite data [17]. Li Ying et al. proposed a sea-surface oil slick identification method combining texture features, principal component analysis (PCA), and directional gradient edge detection method based on the Penglai 19-3 oil spill HJ-CCD data. This method improved the accuracy of oil spill identification [18]. In 2013, Sun Peng et al. analyzed the correlation between oil slick thickness and various spectral indices [19]. Sankaran Rajendran et al. discussed the ability of Snow Water Index (SWI), Normalized Difference Vegetation Index (NDVI) and Normalized Water Index (NDWI) to identify oil spills [20]. Traditional methods mostly used the original spectral reflection signals or spectral indices of HSI to distinguish the surface oil slicks from the background seawater. However, these methods only took advantages of a part of the spectral characteristic signals. They ignored a large number of spectral detail signals.

In order to comprehensively describe the detailed spectral characteristics of oil slicks in HSI and detect oil spills by extracting the key differential information, an advanced spectral DNA encoding (ASDE) method was proposed to carry out the task. The ASDE method was used to detect oil spills in Zhuhai-1 satellite images. First, the original spectral reflection signals of Zhuhai-1 satellite imagery were translated into spectral DNA codeword sequences. Then, the key differential spectral codewords, called spectral genes, of the oil slicks were obtained through two kinds of gene extraction strategies. Finally, the matching rate between encoded pixels and the extracted spectral genes was calculated. The calculation results would be used to produce oil spill detection results. In this research, three Zhuhai-1 satellite images were used to validate the performance of the ASDE method. The experimental results showed that the ASDE method could accurately describe the spectral details of the sea-surface oil slicks in the Zhuhai-1 satellite data. In addition, it can accurately detect the oil spills in the Zhuhai-1 satellite data.

## 2. Materials and Methods

### 2.1. Marine Oil Spill Detection Theory in Hyperspectral Data

**Figure 1:**
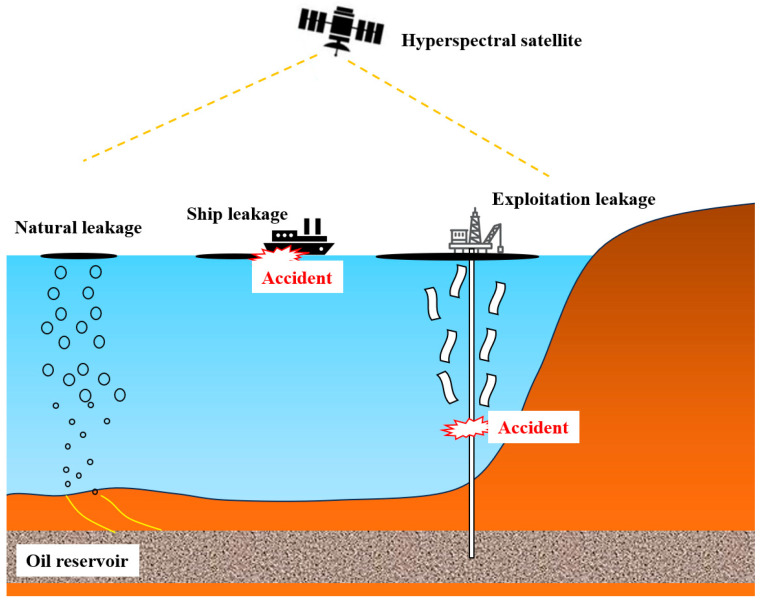
Oil slicks on the sea surface formed by the oil spills.

Marine oil spills are mainly divided into three types: natural leakage from submarine oil reservoirs, crude oil exploitation leakage, and ship leakage (Figure 1). Marine oil spills will form oil slicks on the sea surface. From the thinnest to thickest, oil slicks can be categorized into silvery oil slicks, rainbow oil slicks, metallic oil slicks, discontinuous true-colour oil slicks, continuous true-colour oil slicks, and emulsified oil slicks. Oil slicks of different origins, thicknesses, and degrees of weathering exhibit very different visual, morphological, and spectral characteristic information (Table 1). Hyperspectral satellites can acquire rich spectral reflection signals on the earth’s surface over a wide range. Using the spectral differences to distinguish sea-surface oil slicks and background seawater is an important way to detect marine oil spills.

Zhuhai-1 satellites (Zhuhai Aerospace Microchips Science & Technology Co., Ltd., Zhuhai, China) can acquire hyperspectral imagery of 32 bands on the earth’s surface, with a spectral resolution of 2.5 nm and a spatial resolution of 10 m. The spectral band ranges from 400 nm to 1000 nm, which covers visible light to near-infrared bands. The central wavelengths are 443 nm, 466 nm, 490 nm, 500 nm, 510 nm, 531 nm, 550 nm, 560 nm, 580 nm, 596 nm, 620 nm, 640 nm, 665 nm, 670 nm, 686 nm, 700 nm, 709 nm, 730 nm, 746 nm, 760 nm, 776 nm, 780 nm, 806 nm, 820 nm, 833 nm, 850 nm, 865 nm, 880 nm, 896 nm, 910 nm, 926 nm, and 940 nm. Zhuhai-1 satellites give satisfactory signal-to-noise performances. In common surface environments, its signal-to-noise is around 30 dB. In the complicated surface environments, its signal-to-noise is around 25 dB. It consists of 4 on-orbit satellites (OHS-01, OHS-04, OHS-03, and OHS-04). The revisit period of the satellite constellation is as short as 1 day. Zhuhai-1 satellites can quickly respond to oil spill accidents and provide services for industrial applications such as oil tanker leakage, oil production platform damages, etc. It is an important data platform for marine oil spill identification and monitoring.

### 2.2. Detecting Marine Oil Spills by ASDE Method

**Figure 2:**
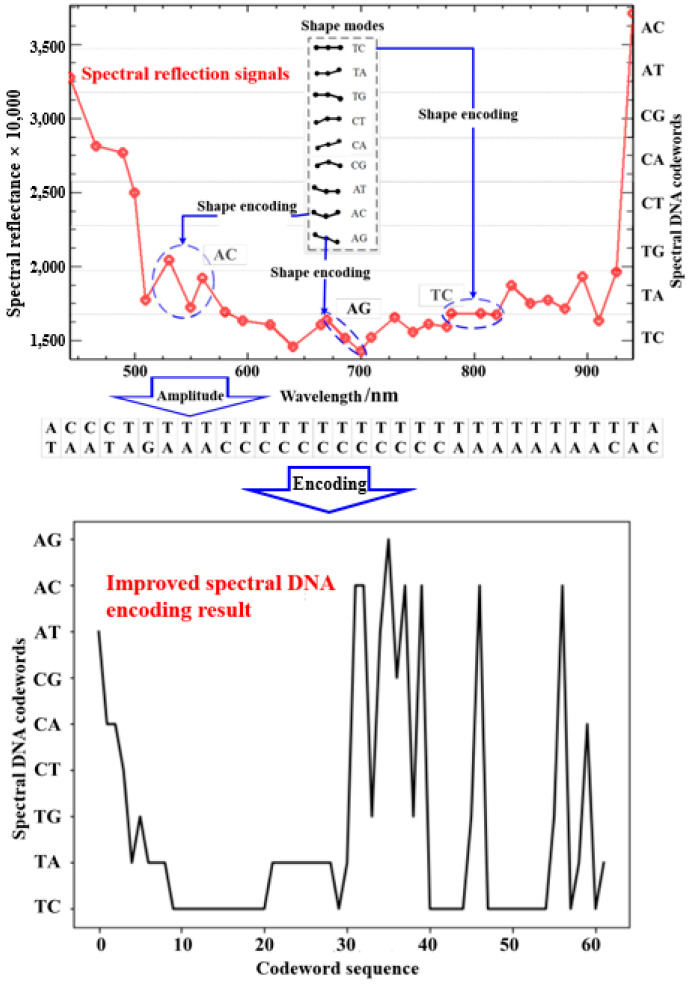
The schematic diagram of the principle of the ASDE method.

The spectral DNA encoding method is an excellent method for describing spectral information. It uses 4 codewords {T, C, A, G} to describe the amplitude of spectral signals. It shows excellent performance for describing spectral characteristics. The spectral feature mining method based on spectral DNA encoding results possesses strong target-discrimination ability [21,22]. However, the traditional spectral DNA encoding method only describes the spectral amplitude information in four intervals. It inevitably misses the detailed differences in spectral amplitudes. In addition, the traditional spectral DNA encoding method describes 9 waveform modes of the spectral shape characteristics through 4 codewords {T, C, A, G}, which causes the codeword ambiguity problem in pattern recognition. To address these problems, an ASDE method is proposed in this research to describe the amplitude and waveform details of Zhuhai-1 satellite imagery. The proposed method contains 3 core procedures:

(1) Divide the amplitude and waveform information of hyperspectral satellite data into 9 modes (Formulas (1)–(6)). Use a pair of DNA codewords to describe the modes of spectral signals (Figure 2).
(2) Use fuzzy clustering algorithm to extract the spectral genetic fragments of the sea-surface oil slicks (Formulas (7) and (8)).
(3) Calculate the similarity between spectral genes and encoded pixels. Extract oil slick objects in the Zhuhai-1 satellite imagery based on the calculation results.
  (1) $$T^{middle}=\frac{\alpha }{N}\sum R_{i},\;\;i\in \left[1,N\right]$$
  (2) $$T_{j}^{upper}=\frac{j\times {\sum R}^{upper}}{4\times N^{upper}},\;\;j\in \left[1,2,3\right]$$
  (3) $$T_{k}^{lower}=\frac{k\times {\sum R}^{lower}}{4\times N^{lower}},\;\;k\in \left[1,2,3\right]$$
  (4) $$Code^{Amplitude}\left(i\right)=Amp\left(T^{middle},T^{upper},T^{lower},R_{i}\right)$$

Equations (1)–(4) describe the amplitude encoding process of the spectral reflection signal of hyperspectral satellite data. The ASDE method uses 7 amplitude thresholds (*T^middle^*, *T^upper^*, *T^lower^*) to divide the reflected signal into 8 intervals. Different DNA codeword pairs are used to describe spectral reflection signals whose amplitudes lie within the interval. *T^middle^* represents the average threshold of the spectral reflection signal. In the formula, Ri represents the spectral reflection signal of the i-th band, and N represents the number of bands. *R^upper^* represents the spectral reflection signal with an amplitude higher than *T^middle^*, *N^upper^* represents the total number of spectral bands with an amplitude higher than *T^middle^*, *R^lower^* represents the spectral reflection signal with an amplitude lower than *T^middle^*, and *N^lower^* represents the total number of spectral bands with an amplitude lower than *T^middle^*. *α* is an adaptive parameter of amplitude encoding, which is used to adjust the distribution range of amplitude symbols. The larger the value of *α*, the more detailed the description of high reflectivity signals. Conversely, the more detailed the description of low reflection signals. When the original spectral signal is distributed among the code element blocks delineated by the amplitude threshold, the amplitude signal of the band is described by the DNA codeword pair corresponding to the code element interval.

(5) $$\Delta =\beta \times \frac{\sum \left(R_{j+1}-R_{j}\right)}{N-2},\;\;j\in \left[1,N-1\right]$$

(6) $$Code^{Shape}(k)=Ptn\left(\Delta ,R_{k},R_{k+1},R_{k+2}\right),\;\;k\in \left[1,N-2\right]$$

Equations (5) and (6) describe the waveform encoding process of the original spectral signal. First, calculate the waveform change gradient Δ of the original spectral signal. Then the waveform mode (Figure 2) is determined based on the gradient relationship of the reflected signals in three adjacent bands. Finally, the waveform information of the spectral reflection signal is converted into DNA codeword pairs according to the waveform mode. *β* is the adaptation parameter of the waveform gradient, which is used to adjust the sensitivity of the waveform encoding. The larger the *β* value, the stronger the tolerance of the spectral codeword to noise. The smaller the *β* value, the stronger the sensitivity of the spectral codeword to noise.

(7) $$Gene(s)=\left\{\begin{array}{l} Code(S),\;\;F(Code(s))\geq \delta \\ 0,\;\;F(Code(s))<\delta \end{array}\right.$$

(8) $$Gene(s)=\left\{\begin{array}{l} 0,\;\;\;G(Code(s))\geq \delta \\ Code(S),\;\;G(Code(s))<\delta \end{array}\right.$$

After the spectral encoding process, the spectral genes in the DNA code chains of seawater and oil slick samples are extracted through fuzzy clustering method. In this research, two spectral gene extraction strategies are used: the largest intraclass similarity (LIS) strategy (Equation (7)) and the largest interclass difference (LID) strategy (Equation (8)). The LIS strategy extracts DNA codewords with high frequency (>*δ*) in similar samples as the spectral genes for these types of objects. The LID strategy extracts specific DNA codewords (frequency < *δ* in other categories of samples) as the spectral genes for these types of objects.

**Figure 3:**
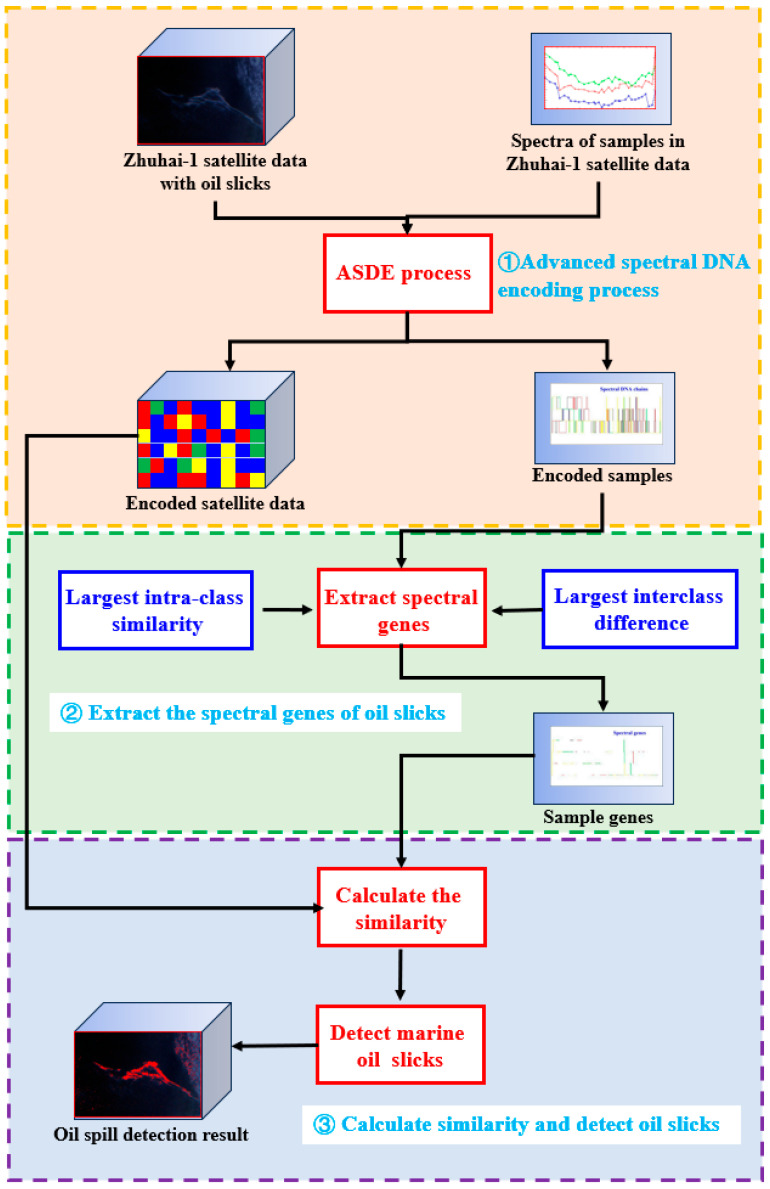
Flow chart of oil spill detection by the ASDE method utilizing Zhuhai-1 data.

Finally, the extracted spectral genes are used to match the encoded satellite imagery pixels. The marine oil spills and background seawater are distinguished based on the degree of matching. Figure 3 shows the process of detecting oil spills in Zhuhai-1 satellite imagery using the ASDE method.

## 3. Results

Three Zhuhai-1 satellite images were used to validate the marine oil spill detection ability of the ASDE method. Since the original satellite data was too large, the oil spill areas were selected for the experiments. Table 2 exhibits the experimental data information. The experimental data were collected from different sea areas. It was beneficial to verify the applicability of the proposed method. It should be stated that, although the marine oil slicks were dynamic, ground truth was produced to evaluate the performance of the proposed method. In this research, manually selected samples were adopted for calculating oil spill detection accuracy like overall accuracy (OA). The oil spill detection performance was evaluated directly. The results were produced by our Python 3.12 codes.

**Figure 4:**
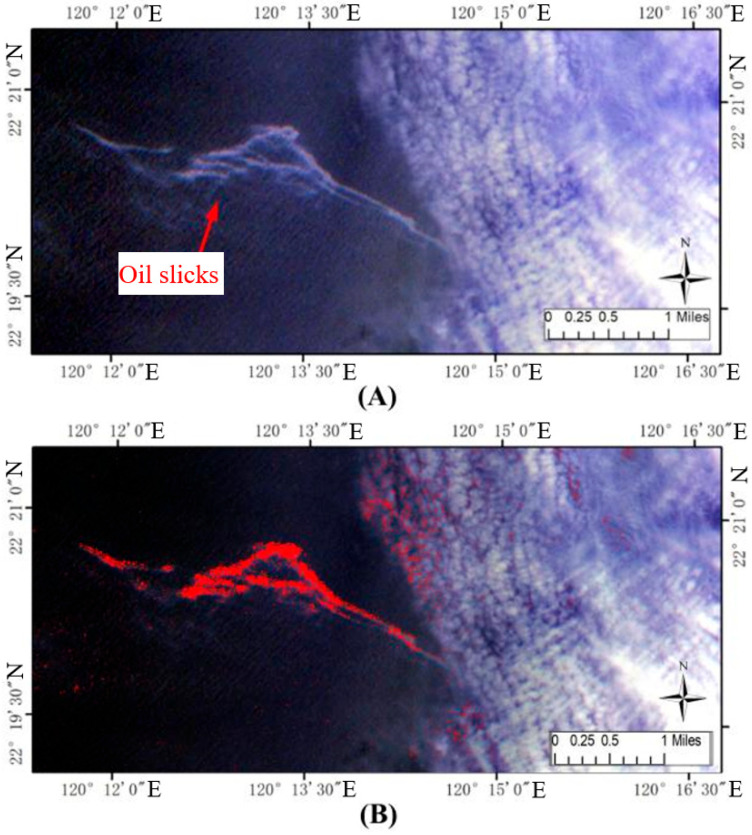
Oil spill detection result of experimental data 1. (**A**) Original satellite image; (**B**) Oil slick detection result.

Figure 4 shows the true-colour composite image of experimental data 1 (Figure 4A) and its thematic map of oil spill identification result (Figure 4B). The result was produced by the parameters of *α* = 0.7, *β* = 1.0, and *δ* = 0.6. It should be stated that the optimal parameters were selected by iteration experiments and visual inspection. The study area (9.28 km × 4.43 km) was located in the Tainan Basin which was rich in oil and gas reserves. Oil slicks produced by natural oil spills had been observed many times. The oil slick in this study area was located at the edge of the cloud layer. It was distributed in a ribbon-like shape with local rainbow visual characteristics. The appearance was consistent with the typical characteristics of natural oil spills. In the thematic map of oil spill detection results, the oil slicks extracted by the ASDE method were marked in red. From the identification results, it could be found that the ASDE method accurately distinguished between marine oil spills and background seawater. Except for a small number of thin clouds that were mistakenly identified as oil slicks, most of the natural oil spills were correctly identified.

**Figure 5:**
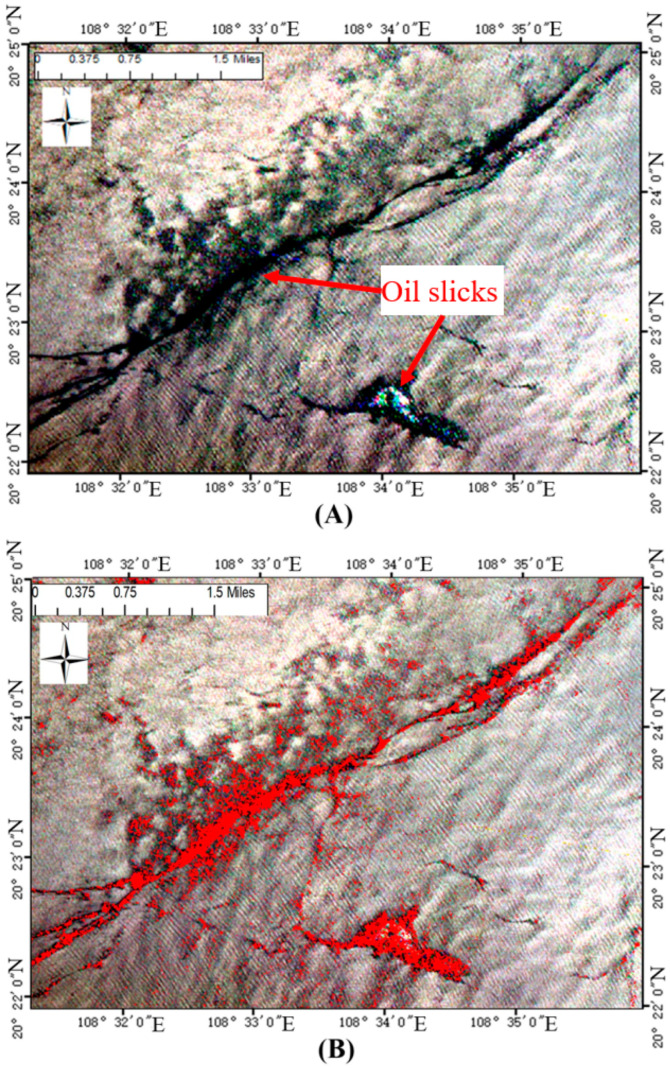
Oil spill detection result of experimental data 2. (**A**) Original satellite image; (**B**) Oil slick detection result.

Figure 5 showed the true-colour composite image of experimental data 2 (Figure 5A) and its thematic map of oil spill identification results (Figure 5B). The result was produced by the parameters of *α* = 1.0, *β* = 1.0, and *δ* = 0.3. During the experimental process, the optimal parameters were selected by visual inspection. The study area (8.10 km × 7.51 km) was located in the Yinggehai Basin. It was surrounded by numerous oil production platforms. Oil slicks caused by accidental oil spills have been observed many times. In this satellite image, the oil slicks were converged in a black stripe. Some emulsified oil appeared white in the core area of oil spill, which only happened when large oil spills occurred. In the detection result, the identified oil slicks by the ASDE method were marked red. Since the surface oil slicks was located in the sun glint area, the seawater presented a higher spectral signal and the shadow region of the waves presented a lower spectral signal. In the detection result, only a small number of shadow areas were misidentified as oil slicks.

**Figure 6:**
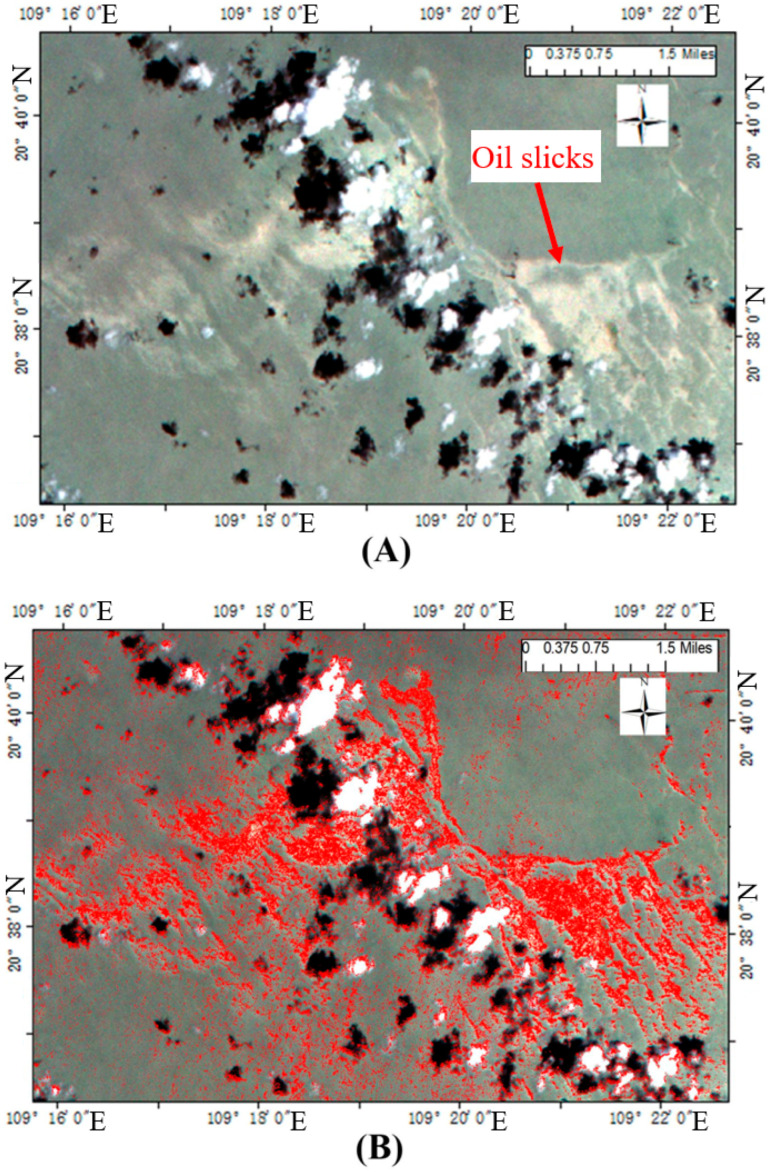
Oil spill detection result of the experimental data 3. (**A**) Original satellite image; (**B**) Oil slick detection result.

Figure 6 showed the true-colour composite image of experimental data 3 (Figure 6A) and its thematic map of oil spill identification results (Figure 6B). The result was produced by the parameters of *α* = 1.0, *β* = 1.0, and *δ* = 0.4. The optimal parameters were selected by visual inspection. The study area (12.04 km × 8.91 km) was located in the Yinggehai Basin. The oil slicks in the study area were diffusive. Affected by the sea wind and ocean current, the oil slicks accumulated on the windward side and spread on the downwind side. In the detection result, the oil slicks were marked red. Since a part of the study area was covered by thick clouds and its shadows, oil slicks in the shadows were not identified. However, the ASDE method detected the oil slicks uncovered by the clouds accurately.

## 4. Discussion

The proposed oil spill detection method based on the ASDE method had two kinds of spectral gene extraction strategies: LIS and LID (formulas 7 and 8). Selecting proper spectral gene extraction strategy was vital for identifying oil slicks in satellite imagery. In addition, the parameters of the spectral amplitude encoding and spectral shape encoding processes would affect oil spill detection accuracy directly. Thus, the spectral gene extraction strategy and the parameters were discussed in this section. In addition, ground truth produced by expert markup was used to validate the performance of the proposed method. Fully connected network (FCN) and support vector machine (SVM) were compares to the ASDE method in this section.

### 4.1. Discussion on Spectral Gene Extraction Strategy

**Figure 7:**
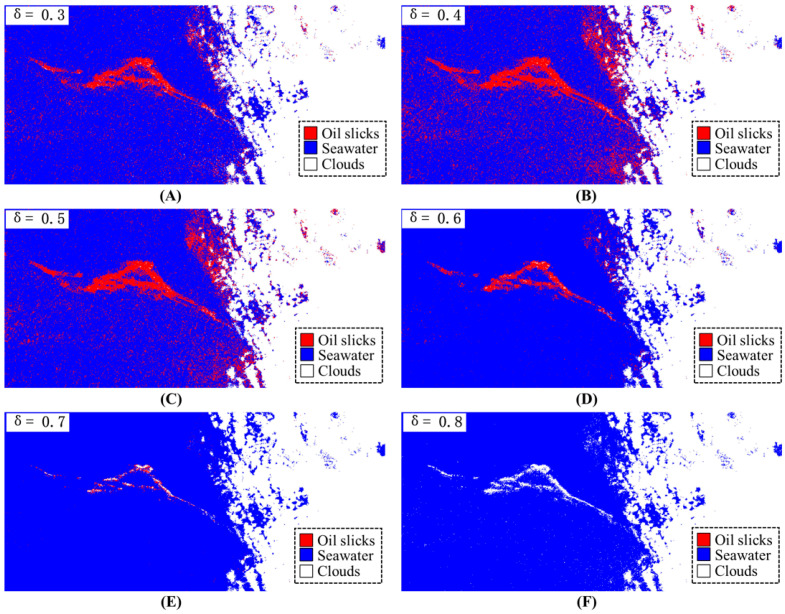
Oil spill detection results produced by the LIS strategy with different *δ*. (**A**) Detection result by *δ* = 0.3; (**B**) Detection result by *δ* = 0.4; (**C**) Detection result by *δ* = 0.5; (**D**) Detection result by *δ* = 0.6; (**E**) Detection result by *δ* = 0.7; (**F**) Detection result by *δ* = 0.8.

When extracting spectral genes using the LIS strategy, high frequency code words (>*δ*) of training samples would be extracted as spectral genes. The size of spectral genes was controlled by the frequency parameter *δ*. Figure 7 showed the oil spill detection results of experimental data 1 with different frequency parameters by LIS strategy (*α* = 1, *β* = 1). In the detection results, the clouds were marked white, the background seawater was marked blue, and the detected oil slicks were marked red, respectively. From the detection results, it could be found that the oil slicks could be detected correctly when frequency parameter *δ* was at a low level (0.3~0.5). Although some seawater was misidentified as oil slicks, the scope of the oil spill was fully identified. It indicated that the ASDE method could describe the spectral details of oil slicks and seawater in Zhuhai-1 satellite data with the LIS strategy.

In the contrast experimental results, when the frequency parameter *δ* increased from 0.3 to 0.6, the oil spill detection performance improved distinctly. It meant that the spectral genes extracted by the LIS strategy could differentiate oil slicks from seawater accurately. In addition, when frequency parameter *δ* increased from 0.6 to 0.8, the oil spill detection performance deteriorated obviously. It indicated that the ASDE method with the LIS strategy needed optimizing in terms of frequency parameters. Selecting proper spectral gene frequency was important for detecting oil spills in different study areas. The best spectral gene frequency was around 0.6 usually.

**Figure 8:**
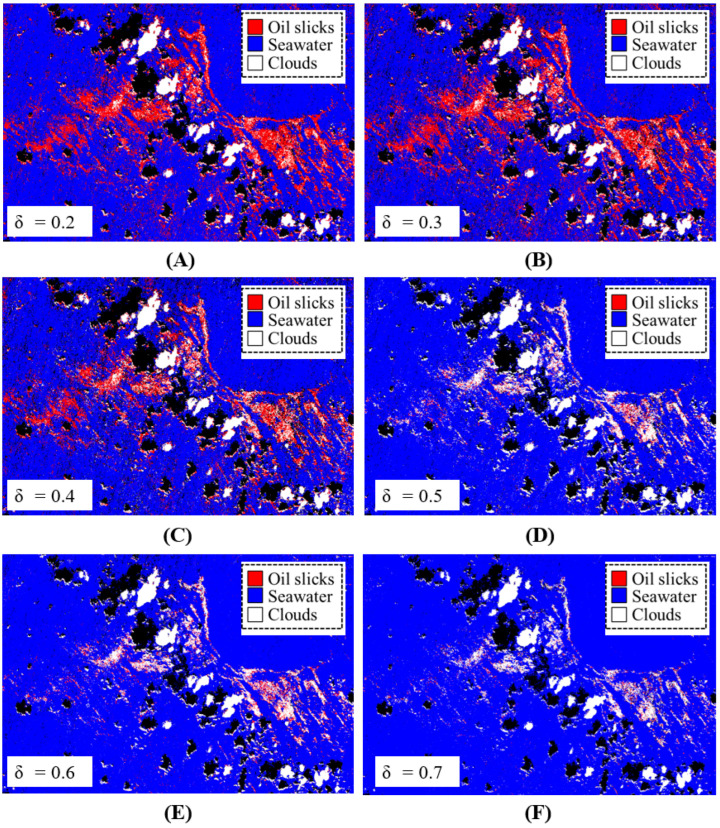
Oil spill detection results produced by the largest inter-class difference strategy with different *δ*. (**A**) Detection result by *δ* = 0.2; (**B**) Detection result by *δ* = 0.3; (**C**) Detection result by *δ* = 0.4; (**D**) Detection result by *δ* = 0.5; (**E**) Detection result by *δ* = 0.6; (**F**) Detection result by *δ* = 0.7.

When extracting spectral genes using the LID strategy, unique code words of oil slicks (spectral gene frequency < *δ* in seawater samples) were extracted as spectral genes. The size of spectral genes was controlled by optimizing the spectral gene frequency *δ*. Figure 8 shows the oil spill detection results of experimental data 3 with different spectral gene frequency using the LID strategy (*α* = 1, *β* = 1). When the frequency parameter *δ* was at a low level (0.2~0.4), the extracted spectral genes were the most unique. It could be found from the results that the oil slicks were identified best when spectral frequency parameter *δ* was 0.3. It indicated that the ADSE method with the LID strategy could extract abundant spectral genes from the Zhuhai-1 satellite to identify oil slicks.

However, in the contrast experimental results, the oil spill detection results got the best performance when the parameter *δ* was around 0.2. It indicated that, although the spectral gene extraction rule was rigorous, Zhuhai-1 satellite data could provide abundant spectral information for differentiating oil slicks from seawater. With the parameter *δ* increasing, the spectral gene extraction rule became relaxed, more and more non-genetic spectral codewords would be extracted for identifying oil slicks. Thus, the oil spill detection performance became worse. From the contrast experimental results, it could be found that the ASDE method could detect oil slicks from Zhuhai-1 satellite data accurately. The ASDE method with the LIS strategy should use a high frequency parameter (*δ* = 0.6) and ASDE method with LID should use a low frequency parameter (*δ* = 0.2).

### 4.2. Discussion on Spectral Encoding Parameters

**Figure 9:**
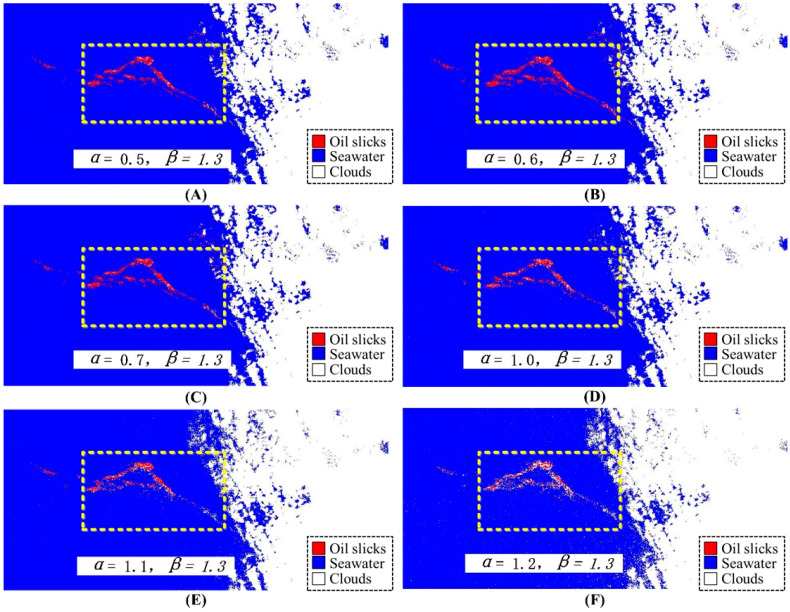
Oil spill detection results produced by different amplitude parameters. (**A**) Detection result by *α* = 0.5, *β* = 1.3; (**B**) Detection result by *α* = 0.6, *β* = 1.3; (**C**) Detection result by *α* = 0.7, *β* = 1.3; (**D**) Detection result by *α* = 1.0, *β* = 1.3; (**E**) Detection result by *α* = 1.1, *β* = 1.3; (**F**) Detection result by *α* = 1.2, *β* = 1.3.

Spectral amplitude encoding parameter *α* and spectral shape encoding parameter *β* were used to adjust the encoding results of the ASDE method. The parameters affected the oil spill detection performance directly. In order to reveal the effect of spectral amplitude parameter *α* on oil spill detection, experimental data 1 was used to conduct contrast experiments (LIS strategy, *δ* = 0.6, *β* = 1.3). Figure 9 showed the oil spill results produced by the ASDE method.

**Figure 10:**
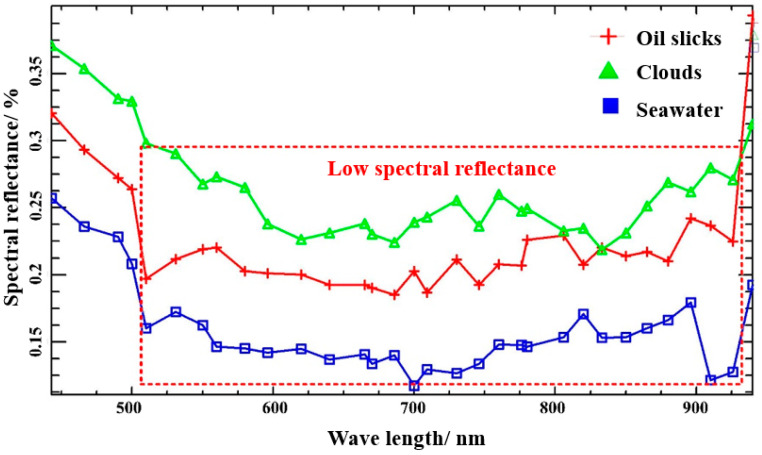
Spectral curves of the oil slicks, seawater, and clouds in experimental data 1.

In the contrast experiments, the spectral amplitude parameter was from 0.5 to 1.5 with the step length of 0.1. Figure 9 showed the most representative results. It could be found from the results that the performance of the ADSE method was satisfactory when the spectral amplitude parameter was small (0.5~0.7). In these results (Figure 9A–C), the identified seawater, oil slicks, and clouds were visually separable. When the spectral amplitude parameter became large (1.0~1.2), the encoding results of oil slicks and clouds were similar. As a result, oil slicks would be misidentified as clouds (Figure 9D–F). Since floating oil slicks had many low spectral reflection signals in Zhuhai-1 hyperspectral images (Figure 10) and small spectral amplitude parameters contributed to describing the low spectral reflection signals, a small spectral amplitude parameter could highlight the spectral differences in oil slicks, seawater and clouds. Thus, it was concluded that the ADSE method could produce excellent oil spill detection result with a small spectral amplitude parameter.

**Figure 11:**
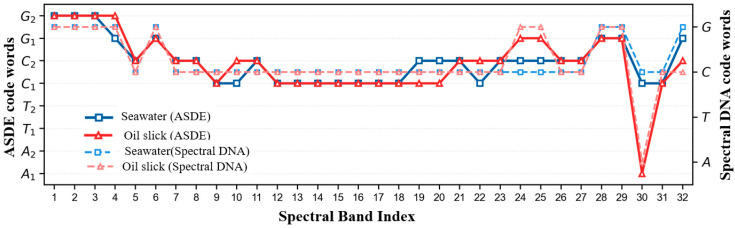
Spectral codewords of the oil slicks and seawater (experimental data 1) produced by the ASDE method and traditional spectral DNA encoding methods.

Figure 11 showed the spectral code words of oil slicks and seawater encoded by the ASDE method and the traditional spectral DNA encoding method. The blue lines were the encoding results of seawater and the red ones were the encoding results of oil slicks. Since the spectral shape code words of the methods were the same, only spectral amplitude code words were exhibited. From the figures, it could be found that, although seawater and oil slicks had low spectral signals, the ASDE method extracted more different code words to distinguish them. It validated that the ASDE method was better than traditional spectral DNA encoding methods for spectral information expression.

**Figure 12:**
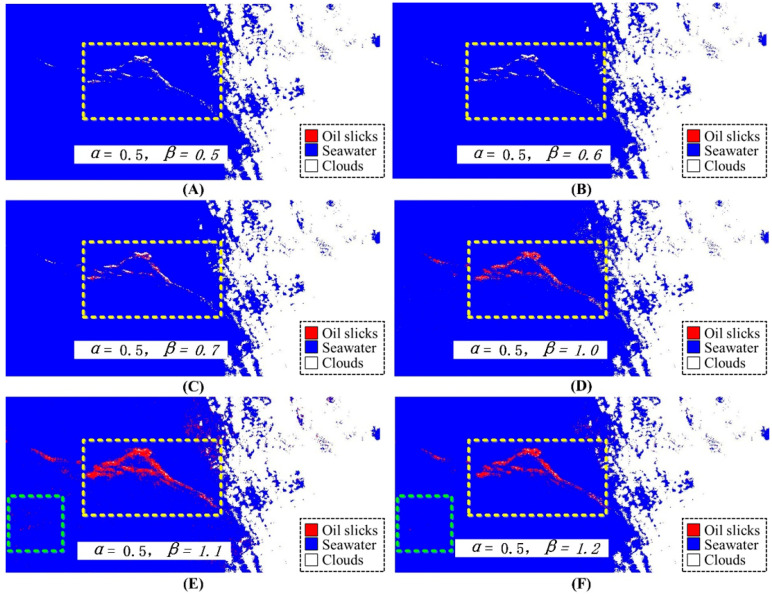
Oil spill detection results produced by different shape parameters. (**A**) Detection result by *α* = 0.5, *β* = 0.5; (**B**) Detection result by *α* = 0.5, *β* = 0.6; (**C**) Detection result by *α* = 0.5, *β* = 0.7; (**D**) Detection result by *α* = 0.5, *β* = 1.0; (**E**) Detection result by *α* = 0.5, *β* = 1.1; (**F**) Detection result by *α* = 0.5, *β* = 1.2.

In order to reveal the influence of spectral shape parameter on oil spill detection, experimental data 1 was used to conduct contrast experiments (LIS strategy, *δ* = 0.6, *α* = 0.5). Figure 12 showed the experimental results. In the contrast experiments, the spectral shape parameter was from 0.5 to 1.5 with the step length of 0.1. Figure 11 showed the most representative results. It was found from the results that the clouds could not be differentiated from the oil slicks when the spectral shape parameter was small (0.5~0.7). When the spectral shape parameter was large (1.0~1.2), the ADSE method could distinguish oil slicks and clouds accurately. Since marine objects were influenced by seawater usually, their spectral signals appeared as random noises, the oil slicks and clouds existed as unstable spectral signals. A small spectral shape parameter would strengthen the interference to spectral signals. On the contrary, a large spectral shape parameter could filter the instability to some extent. Thus, for detecting oil spills in Zhuhai-1 satellite data by the ASDE method one should use a large spectral shape parameter.

### 4.3. Discussion on Accuracy and Superiority

**Figure 13:**
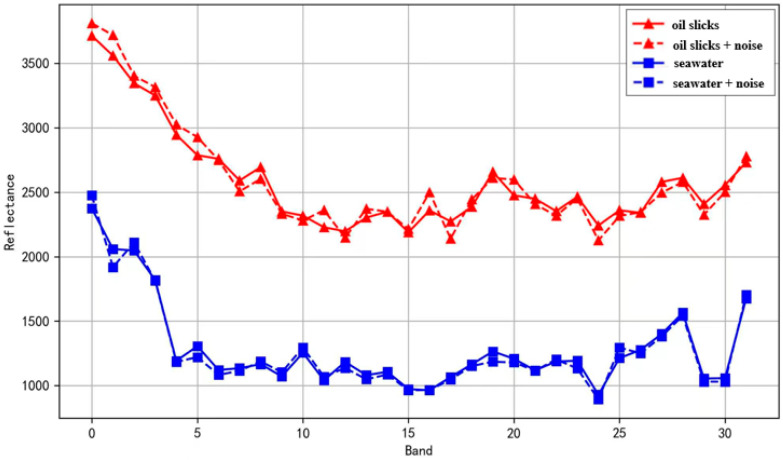
Spectra of oil slicks and seawater of the simulation data.

**Figure 14:**
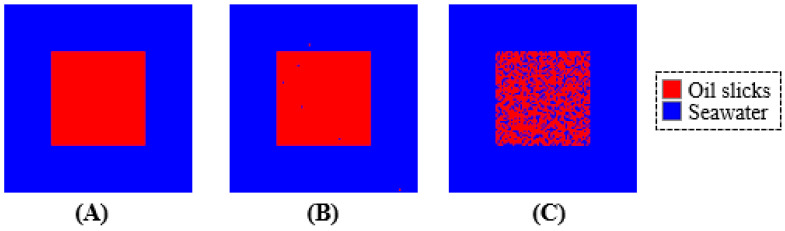
Detection results produced by the ASDE method with the LID and LIS strategies. (**A**) Simulation data; (**B**) Detection result by ASDE method with LID strategy; (**C**) Detection result by ASDE method with LIS strategy.

In order to evaluate the performance of the proposed method quantificationally, a simulation experiment was conducted to validate the detection accuracy of the proposed method. The simulation data was established by spectra of oil slick and seawater. It consisted of 10,000 pixels (100 × 100) including 250 oil slicks and 750 areas of seawater. The spectra of the simulation data were selected from Zhuhai-1 satellite imagery with 3% random noises (Figure 13). The detection results produced by the ASDE method are exhibited in Figure 14.

Figure 14A was the simulation data. Figure 14B was the detection result by the ASDE method with the LID strategy (accuracy = 0.9994, F1 = 0.9988, recall = 0.9984), exhibiting only six misidentified pixels. Figure 14C was the detection result by the ASDE method with the LIS strategy (accuracy = 0.9252, F1 = 0.8241, recall = 0.7008). The simulation experiment indicated that the proposed method could detect oil spills from Zhuhai-1 satellite images accurately.

**Figure 15:**
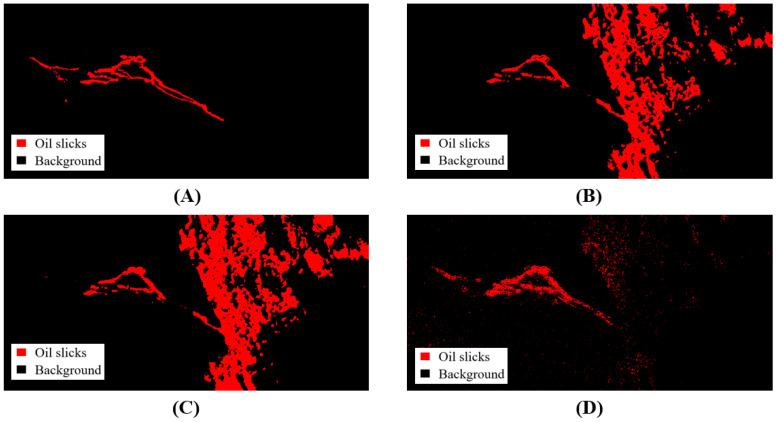
Oil spill ground truth (**A**) and the detection results produced by FCN (**B**), SVM (**C**), and the ASDE method (**D**).

In addition, experiments based on real data were conducted to validate the superiority of the proposed method. The ground truth of experimental data 1 was produced by expert markup (Figure 15A). In the ground truth, oil slick pixels were marked in red. The other objects, such as seawater, shadows, clouds, were marked in black. They were named background. Compared to the original satellite imagery (Figure 4), it could be found that the produced ground truth was consistent with the real oil slick distribution. It meant that the ground truth could be used to evaluate the performance of oil spill detection methods correctly.

In this part, contrast experiments were conducted to evaluate the superiority of the proposed method. FCN and SVM methods were used as contrast (by ENVI 5.1). The oil spill detection results by the compared methods were exhibited in Figure 15. The quantitative metrics of the results were listed in Table 3. According to the experimental results, it could be clearly found that traditional machine learning methods were not suitable for detecting oil slicks in complicated marine environments posed by a small set of training samples. Although FCN and SVM detected the oil slicks with a high overall accuracy (OA), similar pixels like cloud shadow and thin cloud were wrongly detected on a large scale. The ASDE method gained the best OA, F1, and intersection over union (IoU) because it identified oil slicks only by the differential spectral codewords. It avoided misleading signals from the samples with similar spectra. Thus, it performed better oil spill detection accuracy on the Zhuhai-1 satellite imagery. It had to be stated that the machine learning methods tended to be not suitable for detecting objects under the small-sample and similar-spectra environments. It was not degraded.

### 4.4. Shortcomings and Future Work

Marine oil spills produce floating oil slicks on the sea surface. Zhuhai-1 satellite imagery is suitable for detecting marine oil spills. In this research, an ASDE method is proposed to describe the spectral details of oil slicks and seawater. The original spectral signals are translated to DNA code words with nine amplitude elements and nine shape elements. It strengthens the spectral differences in oil slicks, which makes it easy to distinguish oil slicks from seawater and other backgrounds. However, the proposed method has shortcomings: (1) It is a spectral explanatory method. The detection results are pixelated and disperse. The spatial characteristics are not utilized during the detection process. (2) The detection performance relies on experience of selecting proper parameters. It costs a lot of time to produce the optimized results.

In the future, some methods can be used to optimize the method: (1) Combining image segmentation result will increase the oil spill detection accuracy since it takes advantage of the oil’s spatial correlation. (2) The encoded spectral code words can be used as input attributes to establish an artificial neural network. It avoids the need to adjust parameters by hand. (3) It is necessary to amplify the spectral code-word differences. It helps to detect oil spills from the inhomogeneous marine environment.

## 5. Conclusions

Detecting oil spills is important for monitoring marine oil/gas resources and pollution. In order to explore accurate methods for identifying marine oil slicks on hyperspectral satellite imagery, the ADSE method based on Zhuhai-1 satellite data was proposed in this research. The proposed method focused on the fuzziness and ambiguity problems of traditional spectral DNA encoding method. It could describe the spectral details of floating oil slicks. In addition, two kinds of spectral gene extraction methods were used to extract the key spectral differential code words of oil slicks. The extracted spectral genes were used to detect oil slicks by pattern recognition. Experimental results indicated that the ADSE method could detect oil spills in Zhuhai-1 satellite imagery accurately. The ASDE method with LIS should use a frequency parameter *δ* around 0.6 and the ASDE method with LID should use a frequency parameter *δ* around 0.2 to detect oil spills accurately. In addition, the spectral amplitude parameter should be small (around 0.5) and the spectral shape parameter should be large (bigger than 1.0) for Zhuhai-1 satellite data to detect marine oil spills.

## Figures and Tables

**Table 1:** Oil slicks with different thicknesses and their characteristics.

Oil Slicks	Thickness/μm	Bonn Code	Characteristics
Silvery oil slicks	0.04~0.3	Code 1	Silvery oil slicks are silver. Rainbow oil slicks are iridescent. Metallic oil slicks exhibit metallic shine. They are collectively called sheens. Sheens slightly change the colour of the background seawater and is distributed around the thick oil film. Sheens have little spectral difference from the seawater.
Rainbow oil slicks	0.3~5.0	Code 2	
Metallic oil slicks	5.0~50	Code 3	
Discontinuous true-colour oil slicks	50~200	Code 4	Discontinuous true-colour oil slicks are thicker than sheens. They are orange-yellow and distributed in sheets on the sea surface. Their infrared reflection signal is slightly higher than that of seawater.
Continuous true-colour oil slicks	200~500	Code 5	The thickness of continuous true-colour oil slicks is larger than the discontinuous true-colour oil slicks. They distribute in short strips and cannot exist on the sea surface in a large area. They look dark grey or black. Their infrared reflection signal is significantly higher than that of seawater.
Emulsified oil slicks	500~	-	They are weathered oil slicks with huge thickness differences. They look orange-red in colour. They are band-like distributions up to several km in length. They have strong infrared reflection signals.

**Table 2:** Information of the experimental data.

Zhuhai-1 Satellite Data NO.	Shooting Date	Area	Oil Spill Type
HEM1_20210418142028_0008_L1B_CMOS2	17 April 2021	Tainan Basin	Natural oil spill
HCW2_20200722124480_0025_L1B_CMOS3	21 July 2020	Yinggehai Basin	Exploitation accident
HAM1_20210820212223_0018_L1B_CMOS1	19 August 2021	Yinggehai Basin	Exploitation accident

**Table 3:** Oil spill detection accuracy.

Methods	OA	Recall	F1	IoU
FCN	0.87	0.47	0.10	0.05
SVM	0.85	0.56	0.10	0.05
ASDE	0.98	0.54	0.43	0.27

## Data Availability

The data that support the findings of this study are commercial. They are not open dataset. The data can be bought from Aero-Chips (www.myorbita.net).
